# Infective endocarditis after isolated aortic valve replacement: comparison between catheter-interventional and surgical valve replacement

**DOI:** 10.1007/s00392-023-02356-4

**Published:** 2024-01-03

**Authors:** Isabelle D. Ried, Hazem Omran, Max Potratz, Tanja K. Rudolph, Smita Scholtz, Sabine Bleiziffer, Cornelia Piper

**Affiliations:** 1 Clinic for General and Interventional Cardiology/Angiology, Herz- Und Diabeteszentrum NRW, Ruhr-Universität Bochum, Georgstr. 11, 32545 Bad Oeynhausen, Germany; 2 Clinic for Thoracic and Cardiovascular Surgery, Herz- Und Diabeteszentrum NRW, Ruhr-Universität Bochum, Bad Oeynhausen, Germany

**Keywords:** Prosthetic valve endocarditis, TAVI, Surgical aortic valve replacement, Prosthetic heart valves, Endocarditis, Prognosis

## Abstract

**Background and aims:**

Prosthetic valve endocarditis (PVE) is the prognostically most unfavourable complication after aortic valve replacement. This study aims to contribute to a better understanding of the different pathological and therapeutical aspects between PVE following surgical (SAVR) and transcatheter aortic valve replacement (TAVI).

**Methods:**

All patients who had undergone primary isolated SAVR (*n* = 3447) or TAVI (*n* = 2269) at our Centre between 01/2012 and 12/2018 were analysed. Diagnosis of PVE was based on Duke criteria modified in 2015. Incidence, risk factors, pathogens, impact of complications or therapy on mortality were analysed and compared between SAVR- and TAVI-PVE.

**Results:**

PVE incidence did not differ significantly after SAVR with 4.9/100 patient-years and TAVI with 2.4/100 patient-years (*p* = 0.49), although TAVI patients were older (mean 80 vs. 67 years) and had more comorbidities (STS score mean 5.9 vs. 1.6) (*p* < 0.001). TAVI prostheses with polymer showed a 4.3-fold higher risk to develop PVE than without polymer (HR 4.3; *p* = 0.004). Most common pathogens were staphylococci and enterococci (*p* > 0.05). Propensity-score matching analysis showed that the type of aortic valve replacement had no effect on the development of post-procedural PVE (*p* = 0.997). One-year survival was higher in TAVI-PVE patients treated with antibiotics only compared to additional surgical therapy (90.9% vs. 33.3%; *p* = 0.005). In SAVR-PVE patients, both therapies were comparable in terms of survival (*p* = 0.861). However, SAVR-PVE patients who were not operated, despite ESC-guideline recommendation, had significantly poorer one-year survival (*p* = 0.004).

**Conclusion:**

TAVI patients did not have a significantly higher risk to develop PVE. Our data suggest that TAVI-PVE patients in contrast to SAVR-PVE patients can more often be treated with antibiotics only, presumably due to the lack of a polymeric suture ring.

**Graphical abstract:**

Key question:

- What are the differences between SAVR- and TAVI-PVE?
- Are the current ESC guidelines for the treatment of SAVR-PVE also applicable to TAVI-PVE?

Key finding:

- No significantly different PVE incidences after SAVR and TAVI
- Significantly better one-year survival and significantly longer survival in TAVI-PVE treated with antibiotics only compared with additional surgical therapy
- Higher risk to develop PVE after TAVI in patients carrying prostheses containing polymer particles

Take-home message

- TAVI-PVE can often be treated successfully with antibiotics-only, even if surgery would have been indicated according to current guidelines.
- SAVR-PVE patients benefit from surgical therapy, so guideline-compliant surgical indication should be made promptly and performed immediately

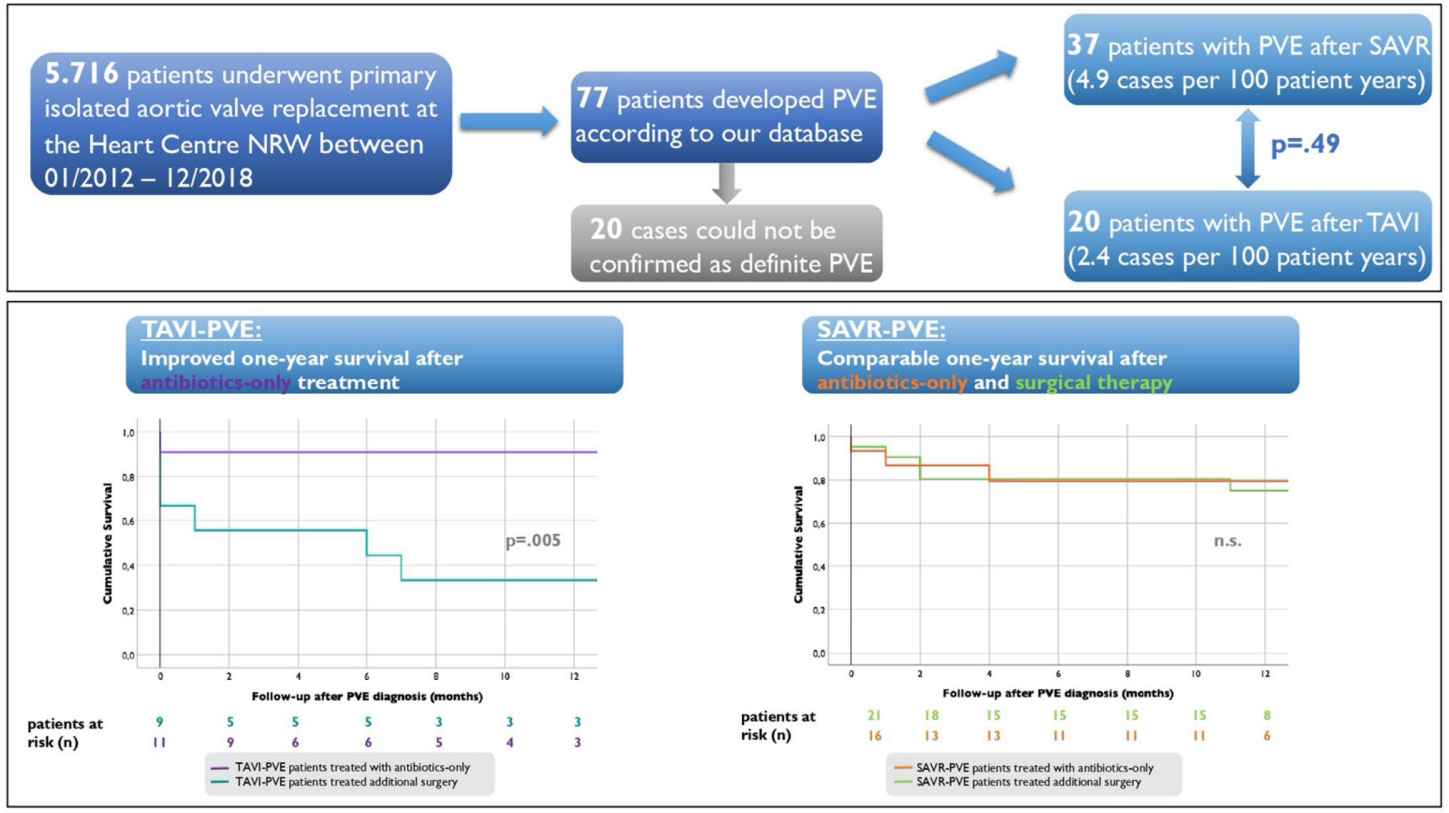

## Introduction

In recent years, the number of isolated aortic valve replacements performed by conventional surgery (SAVR) has been steadily decreasing due to the technological advancement of transcatheter aortic valve replacement (TAVI) and the growing experience of cardiologists [1, 2]. After several large, randomized trials such as the Placement of AoRtic TraNscathetER Valves (PARTNER) -I, -II and -III or the Surgical Replacement and Transcatheter Aortic Valve Implantation (SURTAVI) studies, it has been shown that not only severely ill but also younger and less ill patients benefit from TAVI and that the interventional procedure does not have a negative impact on primary endpoints like mortality and stroke [3, 4]. Since then, TAVI is also indicated for younger patients and those with intermediate and low perioperative risk [3, 5, 6].

Patients undergoing any type of heart valve replacement are ever since at an increased risk to develop a life-threatening microbial inflammation of the prosthesis (PVE) and its surrounding tissues following bacteraemia which are more often seen due to an increasing rate of diagnostic and surgical procedures [7–10]. According to the published data, PVE occurs after SAVR and TAVI with an incidence of 0.4 to 1.9 cases per 100 patient-years [4, 11, 12] and is associated with a high mortality rate of 23–52% [13–16]. As the number of TAVI procedures increases and published data comparing PVE after SAVR and TAVI are limited, we analysed our large patient cohort after isolated aortic valve replacement to identify and compare patients at risk, pathogen microorganisms and the impact on survival depending on complications and treatment strategies after SAVR- vs. TAVI-PVE.

## Methods

### Study design and study population

All patients (*n* = 5716) who underwent primary isolated aortic valve replacement by TAVI or conventional surgery at our Centre between January 1, 2012 and December 31, 2018 were prospectively registered in our database. Follow-up (FU) was performed after 30 days, 6  months, and annually thereafter by means of questionnaires and, if necessary, telephone calls until June 30, 2019. At the cut-off date (June 30, 2019) 5266 of the 5716 patients (92.13%) had (a) returned the FU form which was younger than 6 months and gave information about the absence of an endocarditis or (b) passed away and there was no reference to a possible endocarditis or (c) endocarditis and were therefore considered in our work. In 98% of the patients a FU-form was available regarding mortality.

**Fig. 1:**
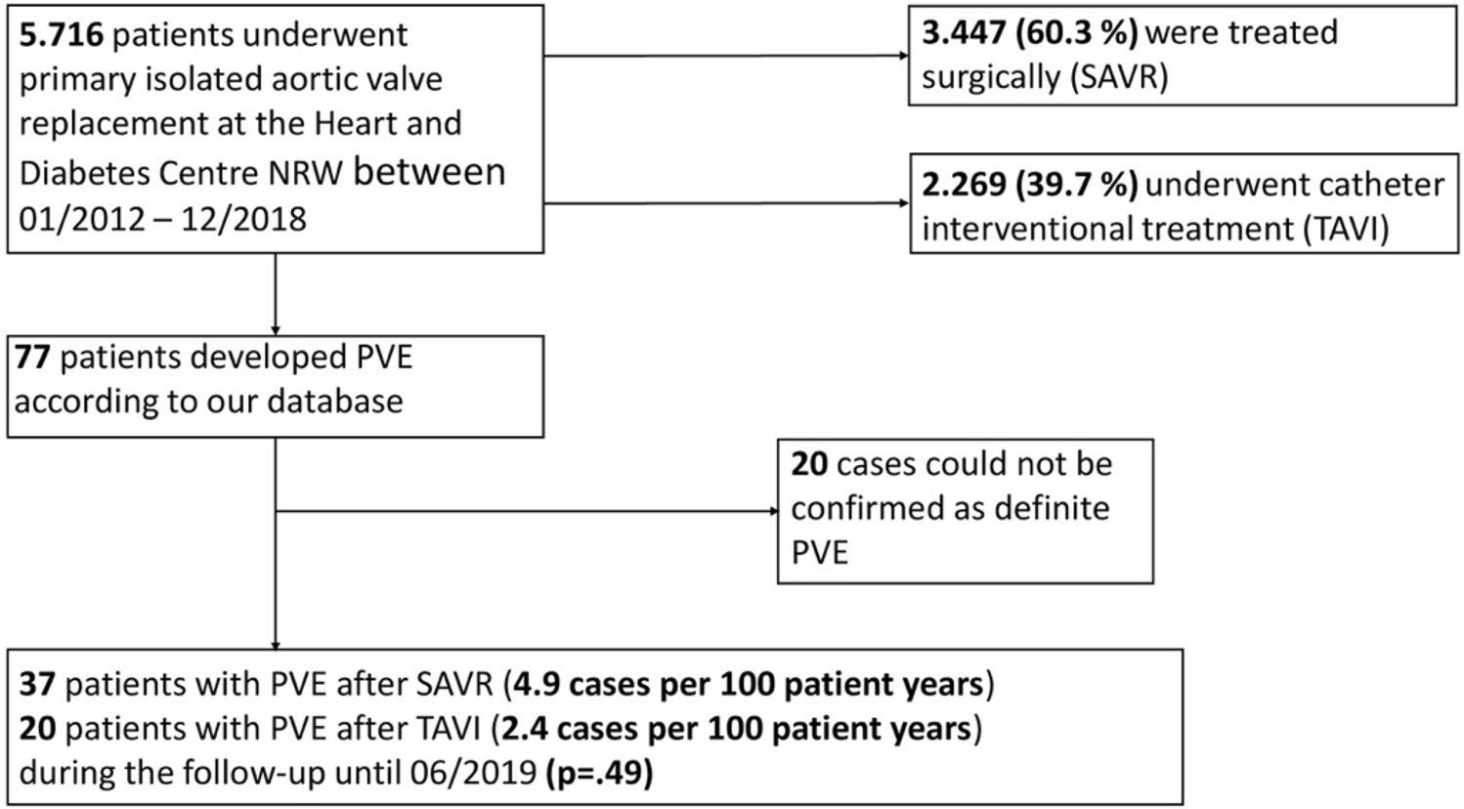
Study cohort gained from our prospective patient database

**Fig. 2:**
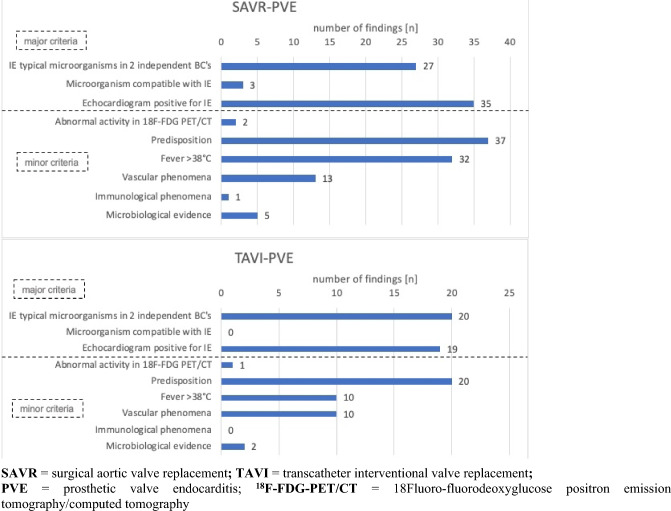
Application of the 2015 Duke criteria in SAVR-PVE vs. TAVI-PVE with specification of the absolute frequencies of the respective major and minor criteria. *SAVR *surgical aortic valve replacement, *TAVI *transcatheter interventional valve replacement, *PVE *prosthetic valve endocarditis, ^*18*^*F-FDG-PET/CT *18Fluoro-fluorodeoxyglucose positron emission tomography/computed tomography

At first, we retrospectively identified 77 prospectively collected patients from the total cohort who developed postprocedural PVE (Fig. 1) according to the data from our database. Thereafter, we applied the 2015 Duke criteria [8] on each patient using the hospital information system, the digital archive and further information from other hospitals such as microbiological findings, medical reports, transthoracic and transoesophageal echocardiography (TEE) findings, and, if available, whole-body PET-CT examinations to confirm the diagnosis of PVE adequately. One third of all patients were diagnosed and treated in other hospitals. Three patients who did not fulfil the 2015 Duke criteria of a definite endocarditis were also included in the analysis because of a relevant clinical picture and an abscess in the TEE. Finally, 57 patients with proven PVE were assigned to our study cohort (Figs. 1, 2).

The study was approved by the local ethics committee (Reg. Nr. 2019-514) and was performed in accordance with the Declaration of Helsinki and the Guidelines for Good Clinical Practice.

### Statistical analysis

Statistical analyses were performed using IBM SPSS version 26 (IBM Corporation Armonk, NY, USA).

Data were expressed as frequencies with percentages for categorical variables and as mean ± standard deviation (SD) with minimum and maximum extreme values for continuous variables. Differences of categorical variables between respective collectives were tested by *χ*^2^ test or Fisher's exact test. For continuous variables, differences between groups with normal distribution were compared using one-way ANOVA. Within-group differences were analysed using repeated-measures ANOVA or paired *t* test. If no normal distribution was found, ANOVA on ranks (Kruskal–Wallis) was performed, and the Wilcoxon signed rank test was used for within-group comparisons. *P* values < 0.05 were considered statistically significant.

Associations were tested using binary linear regression analyses. Univariate and multivariate Cox regression (Cox proportional hazards regression models) were used to test the respective parameters for their prognostic significance with respect to PVE risk and survival probability. Parameters that showed significance levels (*p* < 0.1) in initial univariate analyses were included in a multivariate analysis. Propensity score matching using the “nearest neighbour” algorithm with a caliper of 0.1 was used to match patients receiving either TAVR or SAVR in a 1:1 fashion. The TAVR group was used as the common reference. Matching parameters were COPD, CHD, pAVK, BMI > 30 and preprocedural creatinine. A *p* value < 0.05 was considered significant. Statistical analysis was performed using Statistica 14 (TIBCO, Palo Alto, California, USA) and the R software version 3.6.3 (The R Foundation for Statistical Computing, Vienna, Austria). Survival curves were calculated using Kaplan–Meier curves and the groups were compared with each other using the log-rank test.

## Results

### Incidences of prosthetic valve endocarditis (PVE) and patients’ characteristics

During the FU period from January 2012 until June 2019 with a maximum/mean FU of 89/34 months 57 patients (37 after SAVR and 20 after TAVI) with a proven PVE according to the 2015 Duke criteria were identified. 36 of the 37 SAVR-PVE patients had received biological prostheses and 14 of the 20 TAVI-PVE patients had received prostheses with polymer components: the Edwards Sapiens 3^®^ prosthesis from Edwards Lifesciences^®^ with a plastic skirt made of polyethylene terephthalate (*n* = 13) and the Direct Flow^®^ prosthesis from Direct Flow Medical^®^ with a ring cuff made of polyester instead of a metal cage (*n* = 1).

**Fig. 3:**
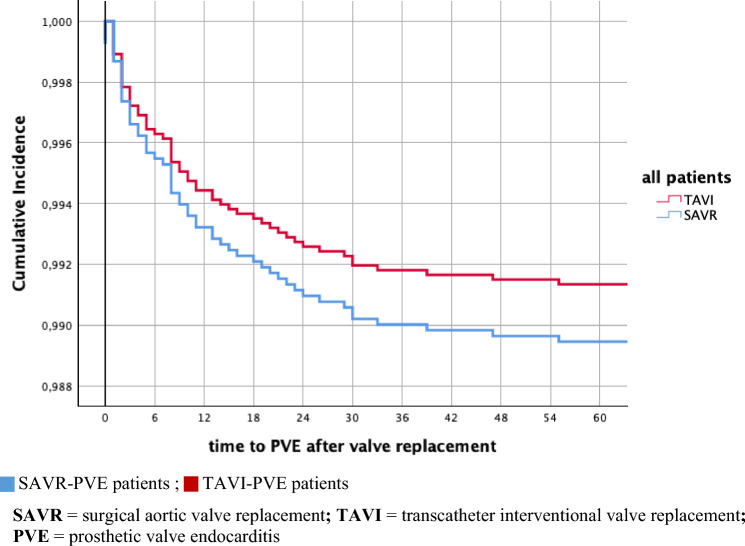
Time to PVE in months after aortic valve replacement: TAVI vs. SAVR. *SAVR* surgical aortic valve replacement, *TAVI* transcatheter interventional valve replacement, *PVE* prosthetic valve endocarditis

After SAVR a numerically higher incidence of 4.9 cases of PVE per 100 patient-years was found than after TAVI with 2.4 cases per 100 patient-years, however without statistical significance (*p* = 0.49). The same holds true for the incidence of early PVE occurring within the first three months after surgery/intervention (3.5 cases/100 patient-years after SAVR vs. 1.9 cases/100 patient-years after TAVI; *p* = 0.683). Overall, patients developed PVE at a mean of 13 months after SAVR and TAVI (*p* = 0.475) (Fig. 3).

**Fig. 4:**
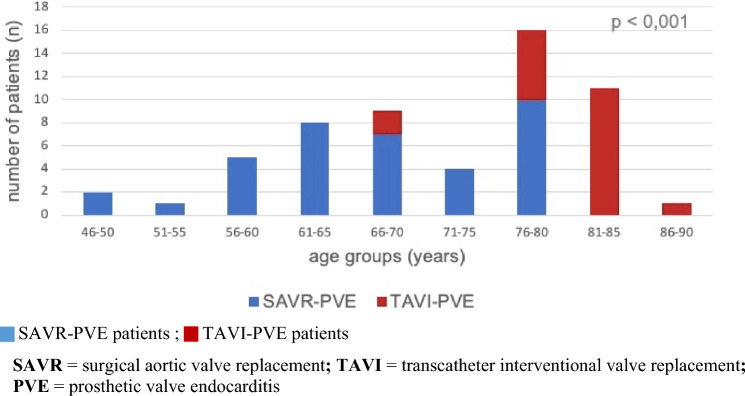
Age groups of the 57 patients with PVE according to treatment of aortic stenosis. *SAVR* surgical aortic valve replacement, *TAVI* transcatheter interventional valve replacement, *PVE* prosthetic valve endocarditis

As expected, PVE patients treated with TAVI were significantly older (mean 80 years) than those treated with SAVR (mean 67 years) (*p* < 0.001) (Fig. 4) and had a significantly higher surgical risk as displayed by the EuroSCORE I, EuroSCORE II and the STS-Score. The baseline characteristics are given in detail in Table 1.

**Table 1:** Preoperative baseline characteristics of patients with PVE

Variable	SAVR (*n* = 37)	TAVI (*n* = 20)	*p* value
Age (years)	67 ± 8.7 [47–80]	80 ± 5.2 [66–87]	< 0.001
Male sex	31 (83.8%)	12 (60%)	0.059
EuroSCORE I	6.3 ± 6.2 [1.5–34.2]	19.4 ± 17.6 [5.5–71.5]	< 0.001
EuroSCORE II	1.6 ± 1.3 [0.5–5.6]	4.4 ± 4 [1.1–16.7]	< 0.001
STS-Score	1.6 ± 0.9 [0.2–4.2]	5.9 ± 4.4 [2–15.1]	< 0.001
Baseline aortic valve area (cm^2^)	0.82 ± 0.2 [0.5–1.5]	0.8 ± 0.16 [0.6–1.3]	0.971
AV: *P*_max_ (mmHg)	74.4 ± 21.6 [27–134]	69.8 ± 22.5 [37–138]	0.456
AV: *P*_mean_ (mmHg)	45.4 ± 12.6 [17–76]	42.8 ± 16.6 [21–98]	0.512
High grade AR (II–III°)	2 (5.4%)	0 (0%)	0.536
LVEF (%)	57.9 ± 8.2 [34–75]	52.1 ± 6.3 [34–60]	0.007
NYHA II	21 (56.8%)	7(35%)	0.117
NYHA III–IV	11 (29.7%)	13 (65%)	0.010
Coronary heart disease	13 (35.1%)	10 (50%)	0.275
Cerebral arterial disease	3 (8.1%)	5 (25%)	0.114
Peripheral arterial disease	1 (2.7%)	6 (30%)	0.006
Atrial fibrillation	6 (16.2%)	6 (30%)	0.309
Pacemaker	2 (5.4%)	5 (25%)	0.084
Stroke	1 (2.7%)	1 (5%)	10.000
Body mass index (kg/m^2^)	30 ± 5.9 [21.2–44.8]	28 ± 5,4 [3–45]	0.283
Diabetes Mellitus_treated_	9 (243%)	4 (20%)	10.000
Renal failure^a^	11 (29.7%)	8 (40%)	0.432
Terminal renal failure^b^	0 (0%)	2 (10%)	0.119
COPD_treated_	0 (0%)	5 (25%)	0.004
Hypertension	29 (78.4%)	19 (95%)	0.139
Cortisone, preoperative	0 (0%)	3 (15%)	0.039

*SAVR* surgical aortic valve replacement, *TAVI* transcatheter interventional valve replacement, *EuroSCORE I & II* European System for Cardiac Operative Risk Evaluation, *STS-Score* Society of Thoracic Surgeons risk score, *P*_*max*_* and P*_*mean*_ maximum and mean pressure gradient of aortic valve, *LVEF* left ventricular ejection fraction, *NYHA* New York Heart Association, *COPD* Chronic Obstructive Pulmonary Disease

^a^Creatinine ≥ 1.2 mg/dl

^b^Dialysis-dependent

### Echocardiographic findings

TEE was performed in 95% (*n* = 53) of all PVE patients. ^18^F-FDGPET/CT was only done in five PVE patients (three SAVR and two TAVI patients). Vegetations were detected by echocardiography in almost all SAVR- (*n* = 32; 86.5%) and TAVI-PVEs (*n* = 19; 95%) (*p* = 0.41). Abscesses were significantly more frequent in SAVR-PVEs than after TAVI (*n* = 16; 43.2% vs. *n* = 3; 15%; *p* = 0.031). All abscesses in TAVI-PVE patients were associated with Edwards Sapiens 3^®^ prostheses which have a so-called plastic skirt made of polyethylene terephthalate.

### Blood culture findings

**Fig. 5:**
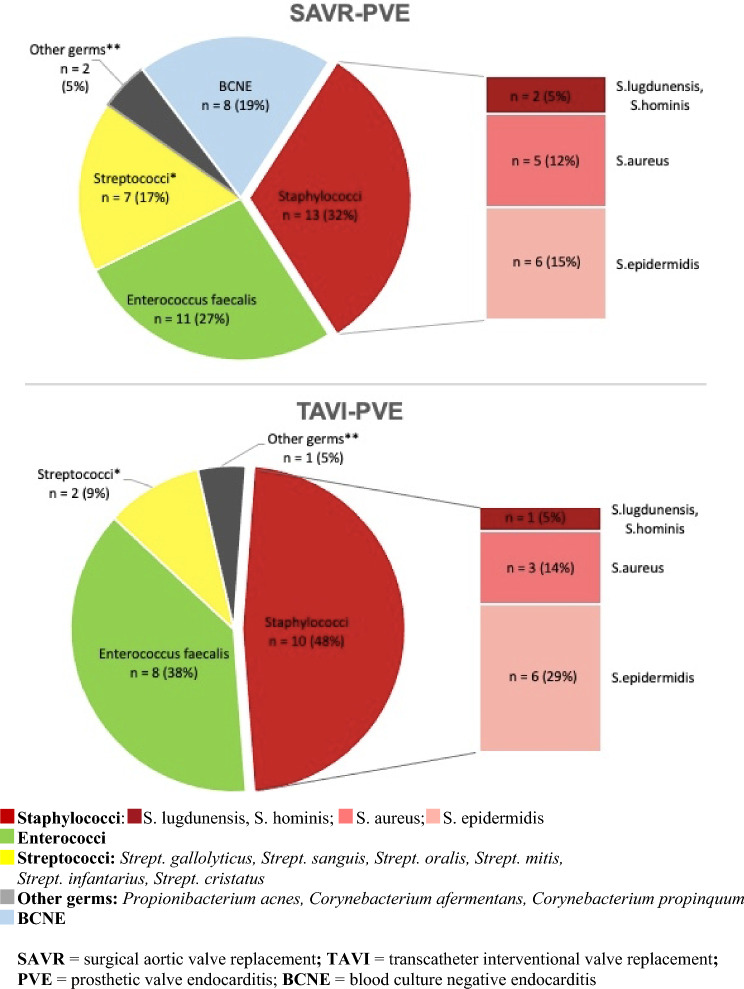
Comparison of PVE causing germs after SAVR and TAVI. *SAVR* surgical aortic valve replacement, *TAVI* transcatheter interventional valve replacement, *PVE* prosthetic valve endocarditis, *BCNE* blood culture negative endocarditis

PVE was most frequently caused by staphylococci in both patient groups after SAVR in 32% and after TAVI in 48% of the cases (*p* = 0.275). *Staphylococcus epidermidis* was not significantly, but more frequently identified in TAVI-PVE (29%) than in SAVR-PVE (15%) (*p* = 0.309). *Enterococcus faecalis* was found second most frequently after both procedures (27% with SAVR-PVE, 38% with TAVI-PVE; *p* = 0.432) followed by streptococci (17% with SAVR-PVE, 9% with TAVI-PVE; *p* = 0.699). In eight (14%) patients with PVE, no pathogen could be identified microbiologically (blood culture negative endocarditis, BCNE). All eight cases of BCNE affected patients after SAVR (19%) (Fig. 5). Staphylococcal PVE resulted in five-fold higher mortality in both treatment groups (HR 5.269; *p* = 0.001) with a significantly better one-year survival for SAVR-PVE patients (53.8%) than TAVI-PVE patients (37.5%) (*p* = 0.047).

**Fig. 6:**
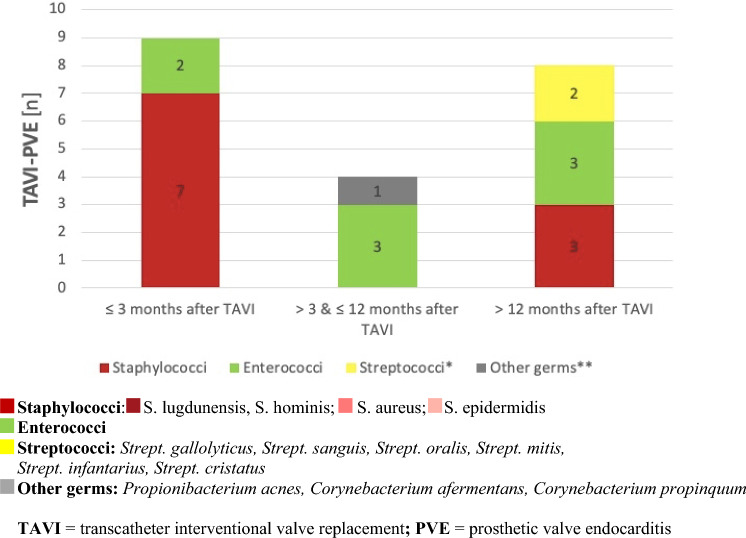
Comparison of PVE causing germs according to early or late onset of TAVI-PVE. *TAVI* transcatheter interventional valve replacement, *PVE* prosthetic valve endocarditis

**Fig. 7:**
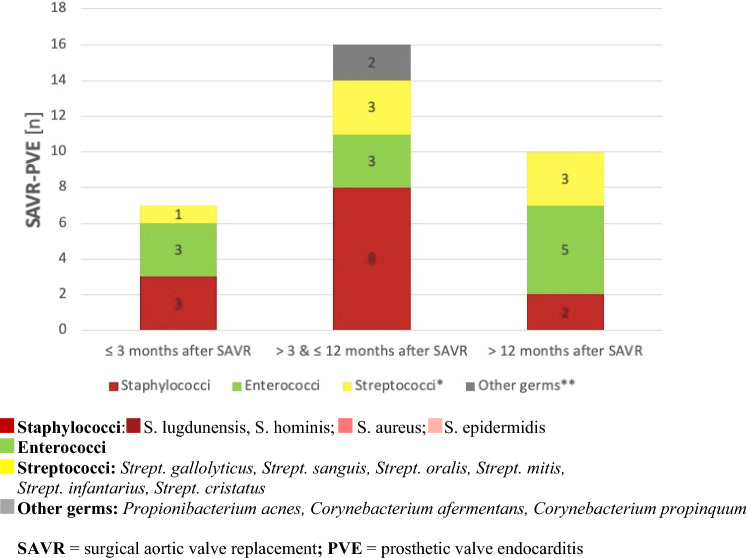
Comparison of PVE causing germs according to early or late onset of SAVR-PVE. *SAVR* surgical aortic valve replacement, *PVE* prosthetic valve endocarditis

Early endocarditis diagnosed ≤ 12 months after valve replacement was mainly caused by staphylococci and enterococci after SAVR and TAVI while late endocarditis diagnosed > 12 months after valve replacement was mainly due to enterococci followed by staphylococci and streptococci, respectively (Figs. 6, 7).

Very early PVE, occurring within the first three months after TAVI, was often caused by staphylococci both after transfemoral (TF) and transapical (TA) access. In contrast, late PVE mainly resulted from enterococci in TF-TAVIs and staphylococci again in TA-TAVIs.

### General risk factors for the development of PVE

Different surgical and interventional parameters like duration of the procedure, need for intubation or intensive care and different intraprocedural complications are listed in detail in Table 2. The different access routes (total vs. minimal sternotomy SAVR; TF- vs. TA-TAVI) did not significantly affect the occurrence of PVE (*p* > 0.05). To identify risk factors for the development of PVE, we first conducted a univariate analysis (Table 3). The parameters that showed significance (*p* < 0.1) in the univariate model were analysed in a multivariate model that was adjusted for the confounders "age", "male gender" and "preoperative risk" (EuroSCORE II) (Table 4). Highly significant PVE predictors in all patients in this analysis were intraprocedural complications (HR 14.866), repeat thoracotomy (HR 3.102) and other infections (HR 5.625). Pacemakers implanted due to post-procedural cardiac arrhythmias did not significantly increase the risk of PVE (*p* > 0.05). The type of procedure (SAVR or TAVI) was not a significant predictor of PVE in either the adjusted multivariate regression analysis or the additional propensity score matching analysis. In the additional propensity score matching analysis, as in the adjusted analysis, other infections (HR 2.065) and periprocedural complications (HR 1.943) were identified as risk factors for postprocedural PVE. In addition, there was a significantly increased risk of PVE after a complete sternotomy in SAVR (HR 1.915). Interestingly, the propensity-score matched analysis showed a decrease in PVE risk with increasing age (HR 0.967).

**Table 2:** Surgical and interventional parameters plus periprocedural complications

Variables	SAVR (*n* = 37)	TAVI (*n* = 20)	*p* value
Incision-suture time (min)	183 ± 43 [112–283]	71 ± 24 [47–150]	< 0.001
Expansion of the annulus	1 (2.7%)	0 (0%)	1
Conversion: TAVI to SAVR	n/a	2 (10%)	n/a
Intubation anesthesia	37 (100%)	7 (35%)	< 0.001
Device misplacement	1 (2.7%)	2 (10%)	0.279
Device embolization	0 (0%)	1 (5%)	0.351
Coronary artery occlusion	2 (5.4%)	0 (0%)	0.536
Pericardial tamponade	2 (5.4%)	1 (5%)	1
LV decompensation	7 (18.9%)	3 (15%)	1
Aortic regurgitation ≥ II°	3 (8.1%)	3 (15%)	0.654
Other intraoperative complications^a^	2 (5.4%)	2 (10%)	0.607
Number of transfused ECs	3 ± 4.3 [0–19]	2.1 ± 4.1 [0–14]	0.194
Length of ICU stay (in hours)	77 ± 149 [10–793]	70 ± 80 [22–398]	0.014

*SAVR* surgical aortic valve replacement, *TAVI* transcatheter interventional valve replacement, *LV* left ventricle, *ECs* Erythrocyte concentrates, *ICU* Intensive care unit

^a^Unstable hemodynamics; right heart failure; tear-off of the right internal mammary artery

**Table 3:** Univariate COX regression analysis to identify significant risk factors for the development of PVE

Metric variables	*p* value	Categorical variables	*p* value
*Preoperative*		*Preoperative*	
Age	0.417	Male sex	0.001
Body mass index	0.025	NYHA II	0.45
HbA1c	0.543	NYHA III-IV	0.336
Creatinine	0.254	Coronary heart disease	0.298
CRP	0.136	Cerebral arterial disease	0.224
Leukocytes	0.961	Peripheral arterial disease	0.161
EuroSCORE II	0.086	Atrial fibrillation	0.673
STS-Score	0.402	Pacemaker	0.079
*Peri-/postoperative*		Stroke	0.884
Incision-suture time	0.609	Obesity^a^	0.305
Ventilation hours	0.373	Diabetes Mellitus_treated_	0.724
Creatinine_maximum_,_postoperative_	0.016	Renal insufficiency^b^	0.215
Red blood cell concentrates	0.004	COPD_treated_	0.676
Stay in hospital	0.361	Hypertension_treated_	0.639
Stay on ICU	0.273	Cortisone Therapy	0.96
		*Peri-/postoperative*	
		SAVR via total sternotomy	0.827
		Transfemoral TAVI	0.909
		Intubation	0.748
		Intraprocedural complications^c^	< 0.001
		Reanimation	0.296
		Extracorporeal membrane oxygenation	0.771
		Rethoracotomy	0.005
		Other infections/inflammations^d^	< 0.001
		New chronic dialysis	0.479

^a^Body Mass Index > 30.5 kg/m^2^

^b^Creatinine ≥ 1.2 mg/dl

^c^Conversion; left ventricular decompensation; haemodynamically relevant pericardial effusion; coronary ostia occlusion; aortic dissection; annulus rupture; aortic regurgitation ≥ II°; device embolisation; acute postoperative dialysis

^d^Wound infection: leg, deep and superficial; mediastinitis; wound infection: thorax, deep and superficial; venous catheter infection; broncho-pulmonary infection; urinary tract infection; bacteraemia/sepsis; peritonitis; systemic fungal infection; oto-laryngological infection; wound healing disorder; recurrent post-thoracotomy syndrome

**Table 4:** Hazard ratios (HR) for factors associated with PVE-adjusted COX regression analysis

Variable	*B*	*p* value	HR	95% CI
EuroSCORE II	0.017	0.806	1.017	0.891–1.161
Body mass index	0.053	0.038	1.055	1.003–1.109
Creatinine_maximum_,_postoperative_	0.181	0.02	1.198	1.029–1.395
Red blood cell concentrates	− 0.143	0.019	0.867	0.769–0.977
Male sex	0.966	0.002	1.008	0.983–1.034
Intraprocedural complications^a^	2.699	< 0.001	14.866	8.502–25.992
Rethoracotomy	1.132	0.005	3.102	1.406–6.843
Other infections/inflammations^b^	1.727	< 0.001	5.625	2.871–11.021

Adjusted for confounders: Age, male sex, preoperative risk (EuroSCORE II)

*B *regression coefficient, *HR *hazard ratio, *95%-CI *95% confidence interval

^a^Conversion; left ventricular decompensation; haemodynamically relevant pericardial effusion; coronary ostia occlusion; aortic dissection; annulus rupture; aortic regurgitation ≥ II°; device embolisation; acute postoperative dialysis

^b^Wound infection: leg, deep and superficial; mediastinitis; wound infection: thorax, deep and superficial; venous catheter infection; broncho-pulmonary infection; urinary tract infection; bacteraemia/sepsis; peritonitis; systemic fungal infection; oto-laryngological infection; wound healing disorder; recurrent post-thoracotomy syndrome

### Effect of TAVI prosthesis type on the development of PVE

Within the TAVI patient population, we investigated to what extent the material, construction and deployment technique of the different TAVI prostheses influenced the risk of PVE.

TAVI procedures were performed from TF in 74% of cases (*n* = 1683) and from TA in 26% of cases (*n* = 586). The access route (TF-TAVI vs. TA-TAVI) showed no significant effect on the occurrence of PVE (*p* = 0.909). 60% (*n* = 1363) of all implanted TAVI prostheses were self-expanding and 40% (*n* = 906) were balloon-expanding. In contrast to the overall collective, 70% (*n *= 14) of TAVI prostheses in the endocarditis collective were balloon-expanding and 30% (*n* = 6) were self-expanding. Balloon-expanding TAVI prostheses thus had a 3.4-fold higher risk of PVE than self-expanding TAVI prostheses (HR 3.44; *p* = 0.013). The TAVI prostheses with polymer structures, namely the Edwards Sapiens 3^®^ prosthesis and the Direct Flow^®^ prosthesis, showed a 4.3-fold higher risk to develop PVE in our cohort than TAVI prostheses without polymer components (HR 4.33; *p* = 0.004). The Edwards Sapien generations investigated in this study (Edwards Sapien 3^®^ and Edwards Sapien XT^®^) did not show a significantly different risk of PVE (*p* = 0.51).

### Treatment of PVE with antibiotics only compared to additional surgery with heart valve replacement

**Fig. 8:**
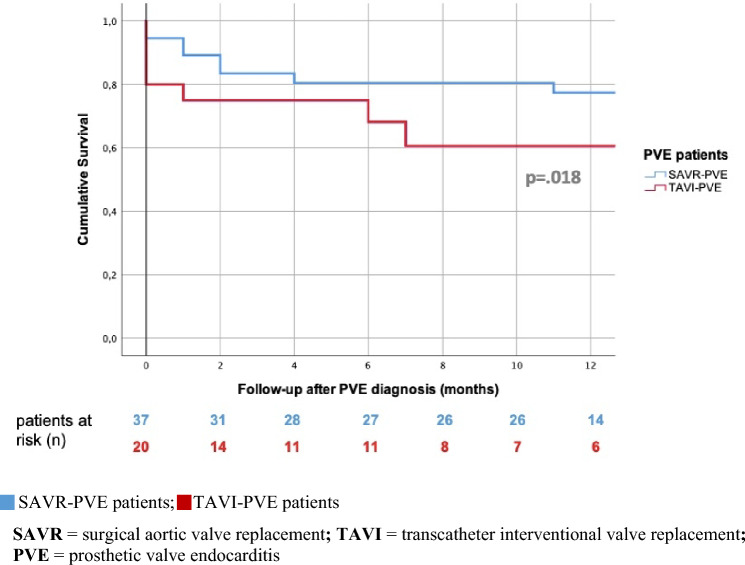
Comparison of unadjusted, cumulative 1-year-survival after PVE between SAVR and TAVI patients. *SAVR* surgical aortic valve replacement, *TAVI* transcatheter interventional valve replacement, *PVE* prosthetic valve endocarditis

In the total cohort, unadjusted one-year survival after the diagnosis of PVE was significantly worse for TAVI patients (60.6%) than for SAVR patients (77.4%) (log-rank: *p* = 0.018) (Fig. 8). These results correspond to the significantly better unadjusted one-year survival of non-PVE patients after SAVR (92.6%) than after TAVI (75.7%) in our cohort (log-rank: *p* < 0.001).

**Fig. 9:**
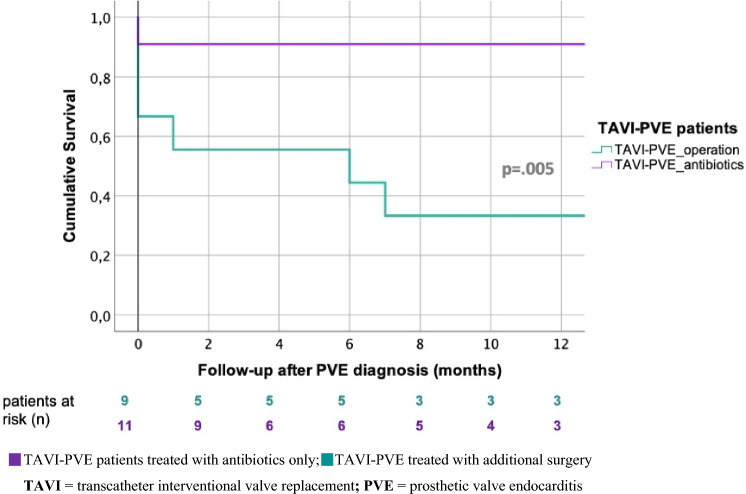
Comparison of unadjusted, cumulative 1-year survival after TAVI-PVE according to treatment: antibiotics-only vs. additional surgical intervention. *TAVI* transcatheter interventional valve replacement, *PVE* prosthetic valve endocarditis

We further investigated the influence of different therapeutic approaches on survival. The unadjusted survival time analysis of TAVI-PVE patients showed that the antibiotic-only therapy led to a significantly better 1-year survival (90.9% vs. 33.3%) and significantly longer survival (36 ± 5 months vs. 9 ± 3 months) compared with additional surgical therapy (log-rank: *p* = 0.005) (Fig. 9). This significantly better prognosis affected both the guideline-compliant and non-guideline-compliant antibiotics-only treatment of TAVI-PVE, regardless of whether TAVI prostheses contained polymer particles or not (85.7% vs. 100%; log-rank: *p* = 0.45) (Table 5).

**Table 5:** Unadjusted survival after TAVI-PVE according to treatment and the effect of polymer components: additional surgical intervention vs. antibiotics-only (guideline compliant vs. not guideline compliant)

TAVI-PVE treatment	TAVI-PVE patients (*n*)	Hospital mortality (*n*)	1-year survival (%)	Mean survival after PVE (months)	*p *(log-rank)
TAVI-PVE_operation_ [prostheses with polymer components^a^]	9 [7]	4^†^ [4^†^]	33.3	9 ± 3	0.005
TAVI-PVE_antibiotics_ [prostheses with polymer components^a^]	11 [7]	1^†^ [1^†^]	90.9	36 ± 5	
ESC guideline-compliant [prostheses with polymer components^a^]	7 [4]	1^†^ [1^†^]	85.7	n.a	0.45
Not ESC guideline-compliant as no surgery was performed [prostheses with polymer components^1)^]	4 [3]	0^†^ [0^†^]	100	n.a	

*TAVI *transcatheter interventional valve replacement, *PVE *prosthetic valve endocarditis, *n.a*. not adjustable

^a^Edwards Sapiens 3^®^ prosthesis with polymeric skirt and Direct Flow^®^ prosthesis with polymeric ring cuff/frame

^†^Dead

**Fig. 10:**
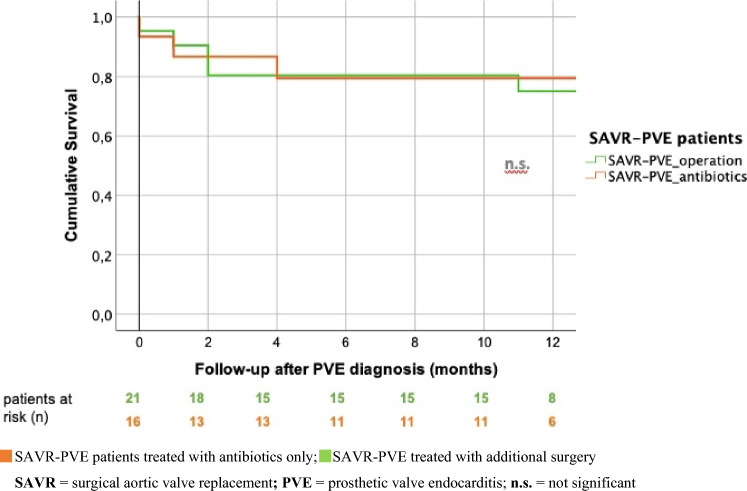
Comparison of unadjusted, cumulative 1-year survival after SAVR-PVE according to treatment: antibiotics-only vs. additional surgical intervention. *SAVR* surgical aortic valve replacement, *PVE* prosthetic valve endocarditis, *n.s.* not significant

On the other hand, the unadjusted survival time analysis of SAVR-PVE patients showed comparable results irrespective of additional surgical therapy in terms of one-year survival (80.8% vs. 75.1%) and overall survival (40 ± 6 vs. 57 ± 9) (log-rank: *p* = 0.861) (Fig. 10). However, regarding ESC guidelines, surgery was withheld in 31% of the SAVR-PVE patients. This not guideline-conform antibiotic-only treatment of SAVR-PVE patients led to a significantly worse one-year survival of 40% compared to 100% in SAVR-PVE patients treated with antibiotics only according to the guidelines (log-rank: *p* = 0.004) (Table 6).

**Table 6:** Unadjusted survival after SAVR-PVE according to treatment: additional surgical intervention vs. antibiotics-only (guideline compliant vs. not guideline compliant)

SAVR-PVE treatment	SAVR-PVE patients (*n*)	Hospital mortality (*n*)	1-Year survival (%)	Mean survival after PVE (months)	*p* (log-rank)
SAVR-PVE_operation_	21	6†	75.1	57 ± 9	0.861
SAVR-PVE_antibiotics_	16	2 †	80.8	40 ± 6	
ESC guideline-compliant	11	0 †	100	49 ± 0	0.004
Not ESC guideline-compliant, as no surgery was performed	5	2 †	40	18 ± 9	

*SAVR *surgical aortic valve replacement, *PVE *prosthetic valve endocarditis

^**†**^Dead

**Fig. 11:**
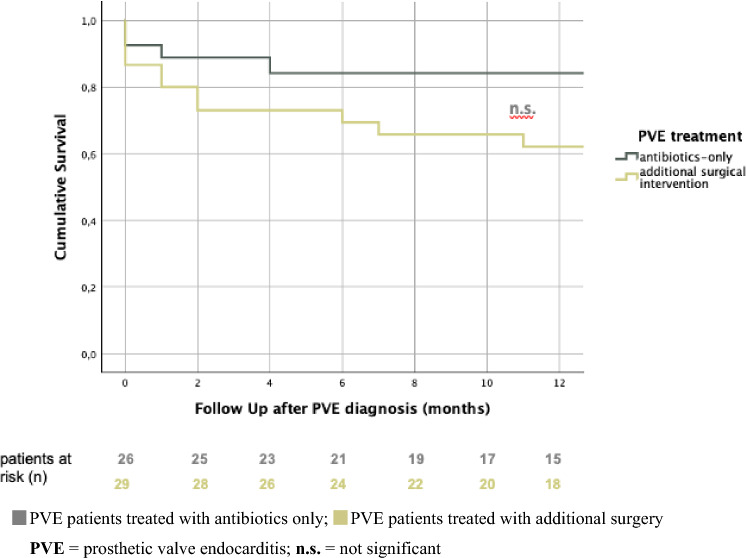
Comparison of unadjusted, cumulative 1-year survival after aortic valve replacement (SAVR + TAVI) according to treatment: antibiotics-only vs. additional surgical intervention. *PVE* prosthetic valve endocarditis; *n.s*. not significant

When comparing the effect of antibiotic-only or additional surgical therapy on survival in the entire endocarditis population, comparable results were seen in terms of overall survival (40 ± 4 vs. 42 ± 8), despite differences in one-year survival (84% vs. 62%) (log-rank: *p* = 0.121) (Fig. 11).

## Discussion

### Main results

The incidence of PVE after TAVI (2.4 cases/100 patient-years) did not differ significantly from the incidence of PVE after SAVR (4.9 cases/100 patient-years). However, in our cohort due to careful follow-up techniques, the overall incidence of PVE was higher than in the literature.

We demonstrated for the first time that TAVI-PVE can often be treated successfully with antibiotics only. In this respect, it has not been discussed so far that the lack of a sewn-in polymeric suture ring in TAVI-PVE prostheses is presumably responsible for this finding.

PVE caused by staphylococci had the worst prognosis with higher mortalities in both treatment groups.

The risk to develop PVE after TAVI is significantly higher in patients carrying TAVI prostheses containing polymer particles.

### Incidence of PVE

In our cohort, with an incidence of 4.9 SAVR-PVE cases per 100 patient-years, twice as many, but not significantly more, patients developed PVE after SAVR than after TAVI, resulting in an incidence of only 2.4 cases per 100 patient-years. In accordance with literature, the assumption that TAVI patients, who suffer more frequently and often more severely from comorbidities thus likely to be exposed to invasive diagnostic and therapeutic interventions with a relevant risk of bacteraemia, will develop PVE more frequently [4, 11–13, 17] could also not be confirmed in our cohort. Furthermore, different procedural techniques resulting from various operative/interventional accesses (transapical vs. transfemoral TAVI l or microinvasive vs. conventional surgery with different length of sternotomy), as well as different kinds of anaesthesia or different length of stay on the intensive care unit between TAVI and SAVR did not result in different incidences of early PVE. The incidences of PVE reported in literature of 0.5–1.86 cases per 100 TAVI patient-years and 0.4–1.9 cases per 100 SAVR patient-years [4, 11, 12, 15] are lower than in our study, but show a wide range due to different inclusion criteria or low case numbers [11]. The relatively high incidence of PVE in our cohort is probably due to the very efficient follow-up techniques in our Centre, resulting in high detection rates of PVE.

### Microbiological profile of PVE

The microbiological profiles in our cohort were different between SAVR and TAVI-PVE as it had been shown previously by Summers et al. [4]. In accordance with literature, staphylococci were the most common pathogens in our cohort of all PVE patients, followed by streptococci and enterococci [4, 7, 12, 18].

Our patients with staphylococcal PVE had the worst prognosis with a 1-year survival of 53.8% for SAVR and 37.5% for TAVI which is in line with the reported 1-year-mortality of 19–48% after staphylococcal PVE in literature [19, 20]. Staphylococcal PVE is a critical disease, which was associated with a five-fold higher mortality risk in our study. In line with the recommendations of Attaran et al., this emphasizes the fact that staphylococcal PVE patients should be treated timely by surgery because they tend to develop early and extensive abscesses. This holds true especially for patients with SAVR prostheses due to biofilm productions at and around the polymeric suture ring, preventing successful therapy by antibiotics only [21].

### General risk factors for development of PVE

Male patients seem to be at a higher risk to develop PVE. In our cohort, men had a slightly but significantly higher risk to acquire PVE, while TAVI-PVE studies by Regueiro et al. and Stortecky et al. reported an almost doubled incidence of TAVI-PVE for men compared to women [15, 22]. A possible explanation might be that men are more likely to suffer from cardiovascular diseases and their complications, thus leading to higher rates of transient bacteraemia and various infections [23]. Furthermore, it has been hypothesized that female hormones are protective against endothelial damage [13].

The need for re-thoracotomy and the occurrence of peri-/postoperative infections were identified as significant risk factors for the development of PVE in our study, comparable to the results of a cohort integrated case–control study by Garrido et al.. They reported that infections or additional surgical re-interventions in the early postoperative period after TAVI or SAVR increased the risk of PVE due to potential bacteraemia with consecutive colonisation of the periprosthetic wound surfaces [24]. This could also be the reason why SAVR patients with complete sternotomy had a higher risk of PVE in the propensity-scored cohort. The fact that the type of aortic valve replacement had no effect on the development of post-procedural PVE was shown in both the overall cohort and the matched cohort. Interestingly, however, we were able to show an inverse relationship between age and the development of post-procedural PVE. This result is of particular interest as predominantly older patients are treated by TAVI while younger patients undergo SAVR. Despite non-significant differences in the risk of endocarditis between the two treatment arms, this could be an indication of a lower risk of infection after TAVI and should be investigated in further studies.

### Effect of TAVI prosthesis type on the development of PVE

Our patients who received a balloon-expanding TAVI prosthesis had a 3.4-fold higher risk of developing PVE than patients who were treated with a self-expanding TAVI prosthesis. The pressure caused by the balloon-expanding prostheses may lead to a greater trauma of the leaflets and the surrounding tissues, favouring the development of PVE through local thrombus and fibrotic formations being superinfected in the context of future bacteraemia. In contrast to our observation, two meta-analyses published in 2019 and 2020, a study using the data from the Infection Endocarditis after TAVI International Register, as well as the analysis of the PARTNER-I and PARTNER-II data, did not show a significantly different risk to develop PVE between balloon and self-expanding TAVI prostheses [4, 16, 17, 25].

Interestingly, TAVI patients in our cohort who had received a TAVI prosthesis with polymeric particles like the Edwards Sapiens 3^®^ or the Direct Flow^®^ prostheses were 4.3 times more likely to acquire PVE during FU than patients with TAVI prostheses without polymer components. It is conceivable that the polymeric skirt of the Edwards Sapiens 3^®^ prosthesis and the polyester-based ring-cuff and framework of the Direct Flow^®^ prosthesis allow gram-positive bacteria to stick to the polymer and to hide from antibiotic treatment. In addition, some staphylococci can evade body's own defence mechanisms and antibiotics treatment by forming a biofilm matrix around themselves and the polymer [26, 27]. We are the first to report about polymer components to be a risk factor for the development of TAVI-PVE, as other studies so far have not looked at polymer particles in TAVI prostheses as a potential risk factor [15, 16]. Our data suggest, however, that patients with high risks of bacteraemia, like diabetics with open wounds due to severe peripheral arterial disease, should preferably not receive polymer-containing TAVI prostheses anymore.

### Optimal treatment strategies of PVE

The decision between an antibiotic-only therapy and the indication as well as the timing for surgical intervention in PVE is a much-discussed topic. The indication for surgical intervention in the often multimorbid TAVI-PVE patients has been very cautious in many centres [14–16, 28–30]. The reluctance to intervene surgically in TAVI-PVE patients is attributed to the high surgical risk in TAVI patients and the surgical challenge of removing an infected TAVI prosthesis [4, 16, 28]

In our study, four of the eleven TAVI-PVE patients treated with antibiotics only should have been operated according to current guidelines for the treatment of infected prosthetic heart valves [8]. Nevertheless, in ten of our eleven TAVI-PVE patients treated with antibiotics only, PVE could be treated successfully. As expected according to the high operative risk, the nine surgically treated TAVI patients of our cohort had a significantly lower 1-year survival (33.3% vs. 90.9%) and a significantly shorter survival after PVE was diagnosed (9 ± 3 months vs. 36 ± 5 months) than the eleven TAVI-PVE patients of our cohort treated with antibiotics only. The studies by Regueiro et al. and Mangner et al. underline the poor outcomes of surgical therapy in TAVI-PVE patients reporting in-hospital mortalities of 30% and 1-year mortalities of 50% in their international TAVI-PVE analysis [15, 16]. In contrast to our data, Mangner et al. did not find better survival rates for patients treated with antibiotics only according to current guidelines [16]. Whether surgical treatment of TAVI-PVE can soon be performed with better results comparable to better outcomes in surgically treated SAVR-PVE patients as more and more TAVI procedures are done in low-risk patients with low comorbidities needs to be proven [4].

In our SAVR-PVE patients, additional surgical therapy as well as antibiotics-only therapy resulted in equal 1-year survival of 75.1% after surgery and of 80.8% after antibiotic-only therapy, while long-term survival was more favourable after additional surgery with a mean of 57 ± 9 months vs. 40 ± 6 months after antibiotics-only treatment in the survival time analyses. The prognosis of the SAVR-PVE patients in our cohort could possibly have been even more favourable if the valid guidelines regarding treatment of infected prosthetic heart valves [8] had been consistently applied to all SAVR-PVE patients. Respectively, the comparison of survival times in our cohort showed that SAVR-PVE patients who did not undergo surgery, despite a given indication according to current guidelines, had a significantly worse one-year survival of 40% compared to a 100% one-year survival of those SAVR-PVE patients being treated according to guidelines with antibiotics only. Our prognostically positive findings emphasize that patients with SAVR-PVE should receive surgical therapy if the indication is given according to the guidelines. This therapeutic handling is strongly supported by the current results of the large EURO-ENDO registry [7].

It is discussed for the first time that this obviously prognostically important difference in the treatment strategy between the benefit of an additional surgical therapy in most SAVR-PVE patients and the preferable antibiotics-only handling in TAVI-PVE patients is related to the presence/absence of a sewn-in polymeric suture ring. In contrast to TAVI prostheses, SAVR prostheses have a suture ring made of polymer, in which bacteria can settle in the course of an infection and partially surround themselves by forming a biofilm. This pathological mechanism is known to prevent successful antibiotic-only treatment of infected polymers, not only in SAVR-PVE but also in patients with infected pacemaker systems in which only an additional total removal of the pacemaker and its leads could cure those patients [8]. Therefore, surgical replacement of the infected heart valve prosthesis is almost always indicated to cure the infection in patients with SAVR-PVE [7, 26]. The finding that TAVI-PVE patients can often be treated with antibiotic-only therapy is probably due to the fact that TAVI prostheses do not have a polymeric suture ring. Polymer particles as found in some newer TAVI prostheses like thin plastic skirts do not seem to have the same pathological effect as thick sewn-in polymeric suture rings of SAVR prostheses with respect to the nesting and protecting behaviours of gram-positive bacteria. It should nevertheless be considered that these results were influenced by selection bias as the endocarditis teams certainly had reasons for not performing surgery. Therefore, statistical bias cannot be ruled out.

## Conclusions

We showed that PVE in TAVI patients can possibly be treated more frequently with antibiotics only, even if surgery would have been indicated according to the guidelines for SAVR-PVE.

We propose that due to a missing polymer suture ring surgery should be applied reluctantly in patients with TAVI-PVE as antibiotic-only therapy often can be very successful. The polymer particles of some newer TAVI prostheses showed no negative influence with respect to antibiotic-only therapy but were associated with an increased risk to develop PVE. Therefore, patients with a high risk of bacteraemia, e.g. diabetics, should probably not be treated with these TAVI prostheses.

On the other hand, SAVR-PVE patients in our cohort, presumably due to the embedding behaviour of the gram-positive bacteria in the polymeric suture ring, showed poor survival without surgical therapy according to the guidelines. In addition, long time prognosis was improved in SAVR patients treated surgically compared to antibiotics-only therapy. Presumably due to the polymeric suture ring, SAVR-PVE patients, benefit particularly from surgical therapy so that the guideline-compliant surgery should be performed immediately to improve the prognosis of SAVR-PVE patients.

In line with the literature, we did not find a higher incidence of PVE after TAVI than after SAVR in our cohort. Furthermore, in the cohort matched for comorbidities, there was no significantly different risk of developing postprocedural PVE between TAVI and SAVR patients. As staphylococci cause foudroyant diseases with a higher risk of mortality, all these PVE patients nonetheless benefit from early surgical therapy.

## Limitation

It should be noted that this work is a monocentric and retrospective analysis with a relatively small collective. With a low number of cases, statistical bias cannot be ruled out. Due to low event rates, the probability of a type II error is increased (low power).

However, due to the very high FU rate of 98% regarding the endpoint mortality, the quality of our results can be rated as high despite the small number of cases in the cohort.

## Data Availability

The data underlying this article will be shared on reasonable request to the corresponding author.
